# Optimization of MBE Growth Conditions of In_0.52_Al_0.48_As Waveguide Layers for InGaAs/InAlAs/InP Quantum Cascade Lasers

**DOI:** 10.3390/ma12101621

**Published:** 2019-05-17

**Authors:** Piotr Gutowski, Iwona Sankowska, Tomasz Słupiński, Dorota Pierścińska, Kamil Pierściński, Aleksandr Kuźmicz, Krystyna Gołaszewska-Malec, Maciej Bugajski

**Affiliations:** Łukasiewicz Research Network-Institute of Electron Technology, Al. Lotników 32/46, 02-668 Warszawa, Poland; gutowski@ite.waw.pl (P.G.); isanko@ite.waw.pl (I.S.); tomasz.slupinski@ite.waw.pl (T.S.); Dorota.Pierscinska@ite.waw.pl (D.P.); Kamil.Pierscinski@ite.waw.pl (K.P.); aleksandr.kuzmicz@ite.waw.pl (A.K.); krystyg@ite.waw.pl (K.G.-M.)

**Keywords:** InAlAs, molecular beam epitaxy, surface morphology, quantum cascade lasers, X-ray spectroscopy

## Abstract

We investigate molecular beam epitaxy (MBE) growth conditions of micrometers-thick In_0.52_Al_0.48_As designed for waveguide of InGaAs/InAlAs/InP quantum cascade lasers. The effects of growth temperature and V/III ratio on the surface morphology and defect structure were studied. The growth conditions which were developed for the growth of cascaded In_0.53_Ga_0.47_As/In_0.52_Al_0.48_As active region, e.g., growth temperature of T_g_ = 520 °C and V/III ratio of 12, turned out to be not optimum for the growth of thick In_0.52_Al_0.48_As waveguide layers. It has been observed that, after exceeding ~1 µm thickness, the quality of In_0.52_Al_0.48_As layers deteriorates. The in-situ optical reflectometry showed increasing surface roughness caused by defect forming, which was further confirmed by high resolution X-ray reciprocal space mapping, optical microscopy and atomic force microscopy. The presented optimization of growth conditions of In_0.52_Al_0.48_As waveguide layer led to the growth of defect free material, with good optical quality. This has been achieved by decreasing the growth temperature to T_g_ = 480 °C with appropriate increasing V/III ratio. At the same time, the growth conditions of the cascade active region of the laser were left unchanged. The lasers grown using new recipes have shown lower threshold currents and improved slope efficiency. We relate this performance improvement to reduction of the electron scattering on the interface roughness and decreased waveguide absorption losses.

## 1. Introduction

Quantum cascade lasers (QCLs) emitting in the mid-infrared are in high demand for such applications as absorption spectroscopy in the molecular fingerprint region [1,2], free space communication [3] and infrared countermeasures [4]. Mostly, they are designed using a InGaAs/InAlAs material system epitaxially grown on InP single crystal substrates, as this group offers wide possibilities of band-gap and wavefunction engineering with high values of band offsets [5]. They consist of the superlattice active region (AR), which is typically built of hundreds of thin layers with thicknesses in the range of few nanometers, and the waveguide layers consisting of a few micrometers of bulk material, both grown in one epitaxial run. A technological feature often observed is that the overall precision of layer thicknesses and their crystalline quality is very hard to achieve at common growth conditions of these two main blocks of different character. In such cases, the main attention is put on strictly required AR superlattice periodicity and thickness control. Epitaxial growth task in MBE is even more complex in the case of strain-compensated QCLs because such QCLs demand a precise control of the composition of four ternary alloys, i.e., InGaAs and InAlAs in AR and waveguide [6]. The optimum MBE growth conditions differ for thin (up to tens or low hundreds of nanometers) and micrometers-thick layers of ternary alloy. The former are the subjects of many literature reports [7,8,9,10,11], as thin films of InGaAs and InAlAs material system on InP substrate are well developed for, e.g., GHz-range transistors or telecommunication lasers, while the thick, high crystalline quality InAlAs alloys required for QCL’s waveguides are rarely studied [12].

The MBE growth conditions which were developed for the growth of In_0.53_Ga_0.47_As/In_0.52_Al_0.48_As active region superlattice of QCL, e.g., growth temperature of T_g_ = 520 °C and V/III ratio of 12, turn out to be not optimum for the growth of micrometers-thick In_0.52_Al_0.48_As waveguide layers. After exceeding ~1 µm thickness, the quality of In_0.52_Al_0.48_As layer deteriorates and formation of microdefects, e.g., Al- or In-enriched clusters or regions of ordered InAlAs alloy on the wafer surface is observed. The in-situ optical reflectometry in MBE shows increasing surface roughness caused by formed microdefects, which is further confirmed by optical microscopy, high resolution X-ray reciprocal space mapping and atomic force microscopy (AFM). We discuss results of diffuse X-ray scattering in InAlAs alloy, which show different features of local chemical clusterring/ordering in crystalline InAlAs alloy depending on MBE growth conditions. AFM pictures show an increasing degree of atomic steps meandering at high thickness InAlAs. As the required waveguide thickness in QCL is at least 2.5 µm, this local chemical clustering/ordering in InAlAs alloy, which can be controlled to some degree in MBE technology, significantly influences the device performance. In this article, based on authors’ efforts to produce lattice matched In_0.53_Ga_0.47_As/In_0.52_Al_0.48_As/InP QCLs with an active region designed for emission wavelength of λ ~9.2 µm, we present optimization of growth conditions of In_0.52_Al_0.48_As waveguide layer leading to the growth of defect free material, with good optical quality. A relatively narrow range of epitaxial conditions for the optimized growth of 2.5 µm thick layers of In_0.52_Al_0.48_As alloy used as the main part of the QCL upper waveguide has been determined. 

The article is organized as follows: in Section 2, the structures and experimental methods used in the studies are described, in Section 3, the experimental results of thick InAlAs layers’ growth versus epitaxial parameters are presented and discussed, including surface morphology studies by optical microscopy, high resolution X-ray diffraction (HR XRD), atomic force microscopy (AFM) and photoluminescence (PL). Finally, in Section 4, the resulting QCL operation with optimized material of thick InAlAs waveguide is presented.

## 2. Materials and Methods

For the purpose of this study, samples were grown in a Riber Compact 21T solid-source MBE, equipped with metal sources for In, Ga and Al, two cells of double-filament type for each metal, and As valved cracker source. The MBE reactor was equipped with in-situ growth diagnostics tools, as pyrometry and optical reflectometry (Laytec) for beam wavelengths of 950 nm, 633 nm and 405 nm. For measurements of substrate temperature, an optical pyrometer (Modline) operating at the wavelength 940 nm was used. The pyrometer was calibrated at oxide desorption temperature from GaAs assumed here as 580 °C, following values of 580–600 °C originally given by Cho [7]. The standard technique, using a beam flux monitor (BFM) located at the growth position, was used for measurements of beam-equivalent pressure (BEP) of molecular fluxes, and reflection high energy electron diffraction (RHEED) was used for in-situ surface studies during growth. Intensities of molecular fluxes, which directly determine the composition of ternary alloys and its growth rates, were calibrated based on high resolution X-ray diffraction measured for each QCL or test sample. Beam flux monitor was used to control day-to-day stability of molecular sources, in particular to set the V/III ratio of arsenic to metal fluxes as one of the epitaxial growth parameters. It should be noted that scaling of V/III ratio between different MBE machines may contain systematic uncertainties related to a particular construction and geometrical configuration of ion-gauge ensemble and may produce different results in different MBE machines even by a factor of a few, e.g., based on our comparison of characteristic points on RHEED surface reconstruction maps for GaAs, which depend on both substrate temperature and V/III BEP ratio [13,14].

The lattice matched QCL structures used in these studies are based on 4-well 2-phonon resonance AR design described in [15]. The waveguide from the bottom side was formed by a low doped InP substrate and from the top by 2.5 μm In_0.52_Al_0.48_As layer covered by a heavily doped In_0.53_Ga_0.47_As layer. The In_0.52_Al_0.48_As waveguide material growth optimization was performed on dedicated test samples grown directly on InP substrates to avoid the influence of another layer growth condition on investigated InAlAs. Besides saving the time and material, it is more important to use samples composed of mainly an InAlAs thick layer, and the interpretation of growth results is more direct than in the case of the whole QCL structure. For this, a simple test structure was drawn up, like the one shown in Table 1. The test structures were grown undoped to avoid any influence of impurities on observed effects.

Series of test samples containing 2.5 μm thick layer of In_0.52_Al_0.48_As, were grown on InP (001) substrates at temperatures in the 460–520 °C range (as measured by pyrometry) and V/III ratio in the 12–31 range. Growth rates ~0.8 μm/h were used for samples in reported studies. In_0.52_Al_0.48_As growth was conducted in As-stabilized conditions (V/III ratio above 10) with surface reconstruction mostly of (2 × 1) or (1 × 1) type as seen by RHEED. Sixteen samples were grown to establish the broad range of important technological parameters and make reliable comparisons.

Samples were characterized using optical microscopy with Nomarski contrast to evaluate the morphology of the surface. High resolution X-ray diffraction (HR XRD) was used to measure lattice parameters and lattice mismatch to InP substrate, chemical composition of alloys, growth rates and reciprocal space maps were recorded to check for a lack of elastic relaxation of layers and to evaluate intensity of diffuse X-ray scattering. Cu Kα_1_, λ = 0.1502 nm radiation was used in triple crystal diffraction geometry [16]. All samples were studied by photoluminescence (PL). The PL spectra were collected by Fourier Transform Infrared (FTIR) spectrometer. For the PL studies, samples were mounted on the cold finger of the LHe continuous flow cryostat and optically pumped with a 650 nm (1.91 eV) laser diode. The PL was collected with an off-axis parabolic metal mirror. The experiment was performed in the step-scan (double modulation) mode. The excitation laser was pulse operated (pulse width of 1.15 ms), with trigger signal referencing the lock-in amplifier. Atomic force microscopy (AFM) was used to evaluate growth features of the surface.

## 3. Results

### 3.1. Background of the Problem

**Figure 1:**
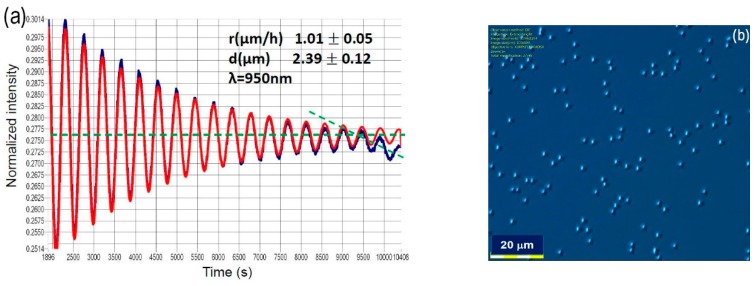
(**a**) comparison of normalized intensity of optical reflection at wavelength 950 nm from the surface of In_0.52_Al_0.48_As sample measured during MBE growth for microdefects-free growth (red line) and for growth with the onset of microdefects and related roughening of sample’s surface in microscale (blue line). Reflectivity was recorded for growth of a 2.5 µm thick In_0.52_Al_0.48_As layer of the upper waveguide on top of the QCL active region. The growth rate was ~1 μm/h; (**b**) Nomarski microscopy picture of surface of 2.5 μm thick In_0.52_Al_0.48_As layer for sample with microdefects developed during MBE growth.

In this part, experimental results on optimization of growth conditions of micrometer-thick In_0.52_Al_0.48_As waveguide layers for InGaAs/InAlAs/InP QCLs will be presented. Considering the standard growth conditions which were developed for growth of AR [6], e.g., growth temperature of T_g_ = 520 °C and V/III ratio of 12, such layer could be grown with high morphological perfection up to about 1 μm thickness, as recognized from our study of the growth of different thickness test samples. After exceeding that thickness, the formation of microdefects (i.e., micrometer size defects) on the wafer surface was observed (Figure 1).

The MBE in-situ optical reflectometry showed increasing of surface roughness manifesting itself as a decrease of average optical reflection signal at growth time around t = 9500 s (Figure 1a, blue line) in comparison to microdefects-free growth (red line). Microdefects were clearly seen on the sample surface in Nomarski microscopy, Figure 1b. It was also noted that the presence of microdefects significantly influenced the QCL device performance by a decrease of optical quality of the waveguide material and increased optical losses. Such microdefects can also decrease the breakdown voltage of dielectric layer deposited during the processing technology of the QCL device. These two reasons have significant influence on limiting the laser performance as well as the device reliability and yield.

**Figure 2:**
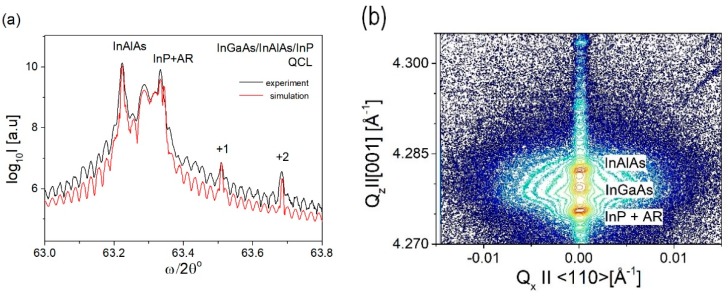
(**a**) ω/2θ scan of QCL sample; (**b**) reciprocal space map of X-ray intensity around 004 Bragg reflection for In_0.53_Ga_0.47_As/In_0.52_Al_0.48_As/InP QCL. Diffuse X-ray scattering is seen as isointensity contours growing in horizontal direction, i.e., perpendicular to [004] Bragg diffraction vector which is displayed in a vertical direction on the map. Subsequent contours of X-ray intensity are shown in a logarithmic scale and separated by a factor of ~3.

An X-ray diffraction profile of In_0.53_Ga_0.47_As/In_0.52_Al_0.48_As/InP QCL, presented in Figure 2a, was used to measure the mismatch of layers to InP substrate and the periodicity of AR superlattice structure. A reciprocal space map of X-ray intensity presented in Figure 2b shows a significant diffuse scattering close to 004 Bragg reflection. It is seen as a spreading of intensity contour lines close to the 004 reciprocal lattice point. It can be seen that the diffuse scattering is located mainly around the InAlAs thick layer’s peak position. Thus, this diffuse scattering is related to microdefects in the InAlAs thick layer. The presence of such diffuse X-ray scattering was seen in QCL samples and test samples with 2.5 μm thick In_0.52_Al_0.48_As layer containing microdefects. Test QCL structures prepared with ~1 μm thick InAlAs waveguide layer showed no X-ray diffuse scattering in reciprocal space maps.

Such microdefects in InAlAs grown close to the lattice-matched condition on InP originate from the presence of aluminum, as they are created only during the growth of InAlAs, but not InGaAs. It will be shown below that, by a careful optimization of growth conditions, e.g., growth temperature and V/III ratio, there is a possibility to minimize their density on the surface, or remove almost all growth-related microdefects. Some random origin microdefects, e.g., the oval defects connected with MBE technology, are still present, but their number is rather small and seems to be independent from MBE growth conditions.

Microdefects in InAlAs/InP described in the literature were reported to depend strongly on the growth conditions, with a rather narrow range of technological parameters of microdefects-free growth [9,12,17] around 500 °C, also with improved InAlAs properties at much higher temperatures around 600 °C [18] or around 650–750 °C used in metalorganic vapor phase epitaxy (MOVPE) [19]. It is, however, difficult to accurately compare various defects reported in relation to growth conditions due to different temperature calibrations used in different growth methods or by different research groups. Our purpose here is to describe defects met in authors’ efforts of developing the technology of microdefects-free thick In_0.52_Al_0.48_As epitaxial layers and relate the microdefects observed to literature reports, based mainly on X-ray scattering studies.

Growth conditions’ optimization was performed with the function of two main epitaxial parameters which are well controlled in MBE—the substrate temperature and the V/III ratio, for a fixed growth rate 0.8 μm for all samples studied. Four different substrate temperatures for growth of test structures were chosen; T_g_ = 520 °C, 500 °C, 480 °C and 460 °C. At each growth temperature, the V/III ratios tested were 12, 17, 26 and 31. Sixteen samples were grown to establish the broad range of important technological parameters and make reliable comparisons. All test samples were designed to have InAlA composition shifted towards slightly higher AlAs composition than In_0.523_Al_0.477_As alloy lattice matched to InP lattice, or to have negative lattice mismatch (grown under weak tensile strain) and $\left|\varepsilon_{⊥}\right|<10^{-3}$. Lattice mismatch was defined, as $\varepsilon_{⊥}=\frac{a_{⊥}(\mathrm{InAlAs})-a(\mathrm{InP})}{a(\mathrm{InP})}$, where $a_{⊥}(\mathrm{InAlAs})$ is lattice parameter of fully strained (in these studies) InAlAs layer in direction perpendicular to InP (001) surface, and a(InP) is lattice parameter of InP substrate. It has been achieved by careful control of lattice mismatch in MBE growth using HR XRD.

**Figure 3:**
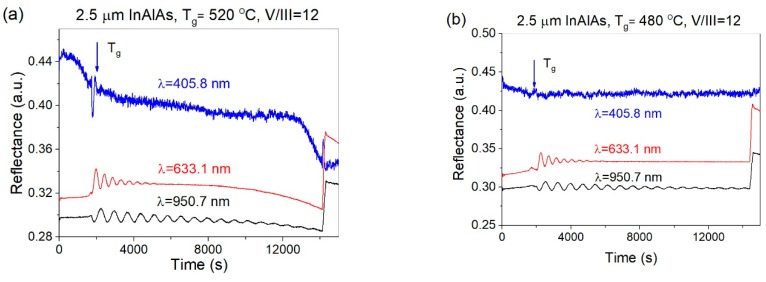
Optical reflectance signals recorded during growth of 2.5 μm thick In_0.52_Al_0.48_As layers: (**a**) T_g_ = 520 °C, V/III = 12 and (**b**) 480 °C, V/III = 12.

During the growth of test structures, the optical reflectometry signal was collected in real time, similarly as described above for QCL growth. Figure 3 shows the comparison of optical reflectance for three different wavelengths; 405.8 nm, 633.1 nm and 950.7 nm, recorded at two substrate temperatures; 520 °C and 480 °C. In the latter case, the substrate temperature was first raised to 520 °C, and then ramped down to 480 °C to start the growth process.

For all wavelengths, the drop of reflectance with time was observed. For quantitative estimation of this effect, we have compared the relative decrease of reflectance at probe wavelength of 633.1 nm at the end of the growth process for all growth conditions investigated. The results are summarized in Table 2. The decrease of reflectance for growth temperature T_g_ = 480 °C is noticeably smaller than for any other substrate temperature. The observed signal drop is related to an increase of surface roughness, due to the forming of microdefects visible on the sample surface, and is in a good correspondence with optical microscopy observations and XRD results, as it will be shown below. However, even for samples showing a significant drop of reflectivity, the surface roughening does not develop at a layer thickness less than ~1 μm, indicating some accumulative mechanism of the onset of microdefects formation.

### 3.2. Surface Morphology

**Figure 4:**
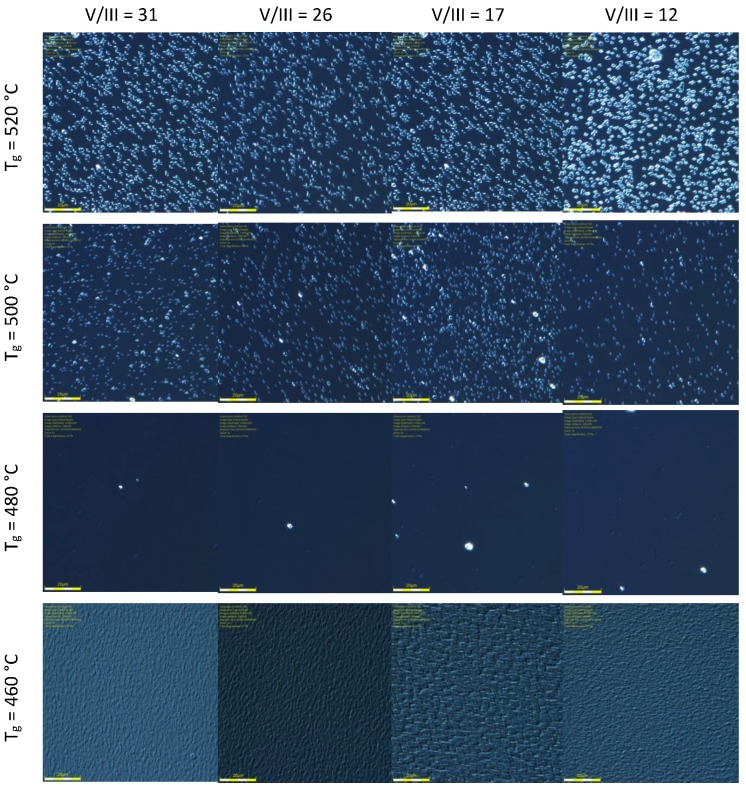
Matrix of optical microscopy pictures (Nomarski contrast) of 0.1 × 0.1 mm^2^ surface area of 2.5 μm thick InAlAs/InP test samples shown versus MBE growth parameters. The rows are for constant substrate temperature and columns are for constant V/III ratio.

Test samples were inspected after the growth process by optical microscope in order to compare surface morphology. The results are presented in Figure 4. Comparing pictures in the columns of constant V/III ratio, a strong dependence is observed in the surface density of growth-related morphological microdefects versus growth temperature. At the same time, comparing pictures in each row, much weaker dependence of microdefects density on V/III ratio is seen. The highest surface densities of growth-conditions-dependent microdefects and the largest size, visible in Nomarski contrast, are present in structures grown at 520 °C. Similar temperatures are used as a standard growth conditions for this material in many reports, e.g., [9,10,12], however usually for thickness well below 1 μm. The reduction of growth temperature to 500 °C (second row in Figure 4) leads to a small decrease of visible microdefects density and size. An almost clean surface is visible for growth temperature in a narrow range around T_g_ = 480 °C (third row in Figure 4). Further temperature reduction to 460 °C leads to a three-dimensional (3D) growth with a dense micro-roughness morphology of a different type than isolated microdefects seen for temperatures 500 °C and 520 °C.

This comparison clearly shows the dominating role of substrate temperature for the development of morphological features in micrometer-scale (microdefects), with only a weak dependence on V/III ratio. In addition, very different morphologies are seen for both of the two highest temperatures and for the lowest temperature. At high temperatures of 520 °C and 500 °C, isolated microdefects distributed on the crystal surface in a close to random manner are seen, while for the lowest temperature 460 °C a dense or continuous micro-roughness is present, with a weak directional texture visible. From a material optimization point of view, it is seen that, in between growth temperature regions of two different types of microdefects, there is a rather narrow window of optimized substrate temperature around 480 °C, probably not wider than ~20–30 °C, which allows for almost microdefect-free growth of high structural and presumably high optical quality InAlAs alloy thick layers, appropriate for the waveguide of QCL. This low concentration of microdefects seen for samples grown at 480 °C is almost independent from the V/III ratio in the range studied. It is seen that the surface temperature during growth plays a more basic role than the V/III ratio (in As-rich conditions used) in controlling of the surface morphology. In the next section, devoted to X-ray studies, we will provide more specific discussion on the crystalline features behind these morphologies.

**Figure 5:**
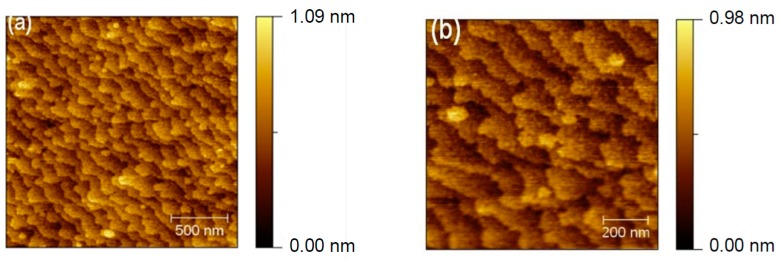
(**a**) AFM picture of surface structure of InAlAs 2.5 μm thick layer grown on InP (001) substrate at the optimal conditions T_g_ = 480 °C, V/III ratio = 26; (**b**) an enlarged picture of the surface. High meandering of atomic steps for growth at this relatively low temperature is visible in spite of low surface density of microdefects developed at such growth conditions as shown in Figure 4.

To learn more about the mechanisms of nucleation of microdefects, we have performed AFM studies for selected samples. Figure 5 shows AFM results of surface structure of sample at optimal growth conditions (T_g_ = 480 °C, V/III ratio = 26). Atomic steps visible on the surface of thick InAlAs layer show distinct meandering, which is characteristic for growth at low temperature and low surface mobility of adatoms. From the meander size of ~100 nm, one may expect that the diffusion lengths of adatoms on the surface is not larger than dozens of nanometers. In spite of such meandered structure of atomic steps, the sample in Figure 4 for conditions T_g_ = 480 °C, V/III ratio = 26 shows very small surface density of microdefects. For samples grown at higher or lower temperatures than the optimum T_g_ = 480 °C, AFM pictures did not show resolved atomic steps which might be consistent with supposed mechanisms of nucleation of microdefects related to local fluctuations of atomic structure at the growth surface. The root-mean-square (RMS) roughness for AFM pictures shown in Figure 5 was 0.14 nm on 10 µm × 10 µm surface.

### 3.3. Structural Characterization

**Figure 6:**
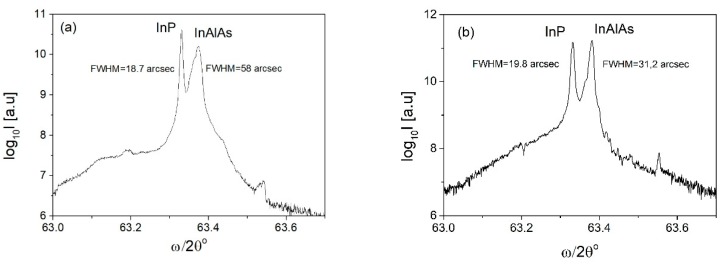
The ω/2θ scans of InAlAs/InP test samples for growth temperatures: (**a**) T_g_ = 520 °C and (**b**) T = 480 °C. V/III = 17 flux ratio was used in both cases.

**Figure 7:**
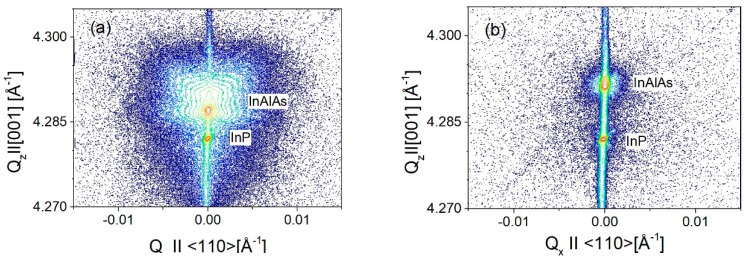
Reciprocal space maps of XRD intensity (contour plots) measured around 004 symmetrical reflection of In_0.52_Al_0.48_As/InP test samples for growth temperatures: (**a**) T_g_ = 520 °C and (**b**) T_g_ = 480 °C. The V/III = 26 flux ratio was used in both cases.

High resolution X-ray diffraction (HR XRD) was used as a standard characterization technique for all samples, to measure the composition of InAlAs alloy, lattice mismatch to InP substrate and layer thickness, while reciprocal space maps were used to evaluate the presence of local lattice distortions and structural fluctuations due to microdefects. Figure 6 shows ω/2θ scans of InAlAs test samples for growth temperatures T_g_ = 520 °C and T_g_ = 480 °C and V/III = 17. A substantial narrowing of InAlAs peak is seen in the sample grown at lower temperature, which is the evidence of better crystalline quality. This observation is further confirmed by the reciprocal space maps of XRD intensity shown in Figure 7. The smple grown at T_g_ = 480 °C shows much less diffuse scattering, comparing to the sample grown at T_g_ = 520 °C.

**Figure 8:**
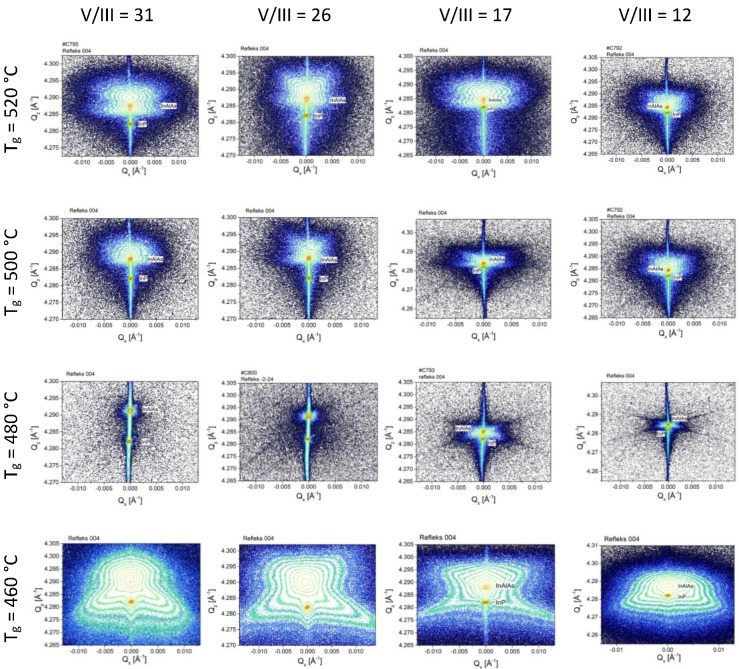
Reciprocal space maps of XRD intensity (contour plots) measured around 004 symmetrical reflection for InAlAs/InP test samples shown versus MBE growth parameters, where rows are for constant substrate temperature and columns are for constant V/III ratio. In the intensity direction, the *z*-axis, the contours are drawn equally spaced in logarithmic scale from the Bragg maxima (~10^6^ cps, counts per second in diffractometer units, to 1 cps background); close contours differ in intensity by a factor of 4. Coordinates of 004 Bragg reflection for InP are approximately (Q_x_, Q_z_) = (0, 4.28) Å^−1^, Q_x_ // [110] and Q_z_ // [001] are reciprocal space vectors, in all panels. Units of scales in Q_z_ directions differ in various panels.

Reciprocal space maps of XRD intensity measured around 004 symmetrical reflection for InAlAs/InP test samples are shown in Figure 8.

At close-to-optimal temperature 480 °C (see Figure 4), a high crystalline perfection of InAlAs layer is seen for V/III ratio not higher than 17, where the diffuse X-ray scattering (DXS) intensity is very low. At higher and lower temperatures, a significant portion of diffusely scattered X-ray intensity around Bragg 004 reflection is visible as widely spread intensity contours. It is seen that the symmetry features of DXS contours in the reciprocal space maps are different for the case of isolated microdefects (520 °C and 500 °C as in Figure 4), where DXS has more isotropic character, and at low temperature (460 °C) where DXS contours elongated in reciprocal space in approximately <111> type directions indicate anisotropic features of microdefects. Distinct anisotropic features are also visible for 480 °C at low V/III ratio = 12, 17, although at low DXS intensity.

Distinct structural features of In_0.52_Al_0.48_As test samples can be seen in X-ray reciprocal space maps (RSM) done as a function of MBE growth parameters. A clear correspondence may be seen of X-ray scattering intensity distributions with the above presented results of in-situ optical reflectance (Table 2) and optical Nomarski microscopy of surface with microdefects (Figure 4). Namely, for samples grown at substrate temperature 480 °C showing low surface density of morphological microdefects, X-ray intensity maps show a narrow distribution concentrated at 004 Bragg reflection maxima for InAlAs layer and InP(001) substrate, as seen in Figure 8, for high V/III ratio of 26 and 31. However, for samples with a high density of surface microdefects, this is going to higher 500–520 °C or lower 460 °C temperatures in Figure 4, a much wider distribution of X-ray intensity scattered around 004 Bragg maximum is visible in respective panels in Figure 8. Such X-ray intensity widely diffusely distributed around reciprocal lattice points (known as diffuse X-ray scattering, DXS) originates from weak deviations of crystal structure from the ideal periodicity [20,21,22,23], or in other words from the structural disorder in crystals or crystalline alloys, and is related to a presence of various structural defects [22] of crystal structure or structural fluctuations in a crystalline alloy [23] (Sections 1.5, 3.2). In Figure 8, DXS is visible as a spreading of intensity contours around 004 Bragg reflection. Summing this up, in Figure 8 going from 480 °C to either higher or lower temperatures, a significant increase of DXS intensity is seen, which is in a good correspondence with an increase of surface density of microdefects in Figure 4.

### 3.4. Photoluminescence

**Figure 9:**
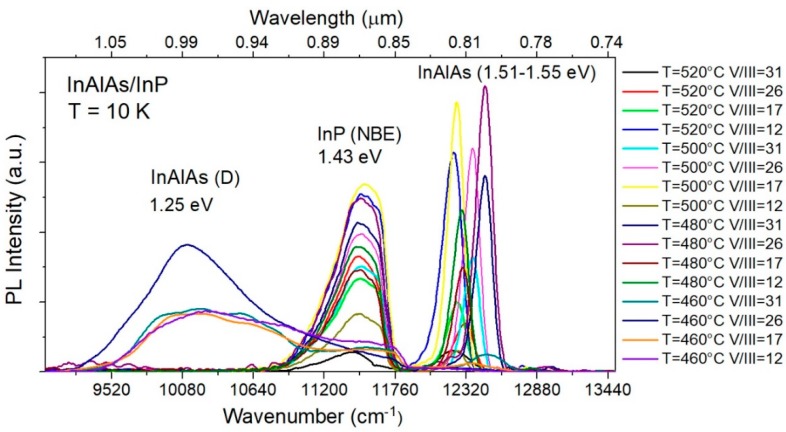
Low temperature PL spectra of In_0.52_Al_0.48_As/InP samples grown at different temperatures and V/III flux ratios. PL was excited by 650 nm (1.91 eV) laser diode.

Photoluminescence spectra of all samples investigated are plotted in Figure 9. For the PL studies, samples were mounted on the cold finger of the LHe continous flow cryostat and optically pumped with a 650 nm (1.91 eV) laser diode.

Three groups of transitions are present in the low-temperature spectra. The lines peaked at ~1.51 eV–1.55 eV can be attributed to near band edge (NBE) luminescence of the InAlAs layer [24,25]. Energy spread of their positions results from differences in alloy’s composition, which were indeed confirmed by X-ray diffraction. The second group of lines, peaked at 1.43 eV, is the near band edge (NBE) luminescence of InP substrate [26]. Both the position and the halfwidth (~50 meV) of these lines correspond well with doping of the substrate (3 × 10^18^ cm^−3^). Weaker long-wavelength band D is also seen around 1.25 eV. It is observed only in material grown at 460 °C to be attributed to defect related emission in InAlAs. Its appearance is accompanied by quenching NBE luminescence. D band is absent in the material grown at optimum growth temperature of 480 °C but is also not seen in high temperature grown InAlAs; i.e., material grown at 500 °C and 520 °C, which are otherwise defected as confirmed by X-ray studies. This means that the nature of the defects generated in both temperature ranges, below and above optimum growth temperature window, is different.

The full width at the half maximum (FWHM) of the near band edge InAlAs PL signal was measured. The results are summarized in Table 3. It shows that, for temperatures close to optimum growth window, as determined from X-ray studies, the NBE peaks are narrowest, which might be directly related to their crystalline quality.

Experiments based on carrier transport indicate that the InAlAs alloy grown by MBE may exhibit significant clustering. Clustering generally has a detrimental effect on the optical and transport properties of materials [11]. Due to the large difference between In-and AI-related bond energies, the In_0.52_Al_0.48_As system is expected to show clustering due to the low temperatures employed in MBE growth [27,28,29,30].

### 3.5. Device Results

The QCL structures were grown using lattice-matched In_0.53_Ga_0.47_As/In _0.52_Al_0.48_As active region. of 4-well 2-phonon resonance design [15]. The layer sequence of one period of the structure, in nanometers, starting from the injection barrier was: **4.0**, 1.9, **0.7**, 5.8, **0.9**, 5.7, **0.9**, 5.0, **2.2**, 3.4, **1.4**, 3.3, **1.3**, 3.2, **1.5**, 3.1, **1.9,** 3.0, **2.3**, 2.9, **2.5,** 2.9 nm. The InAlAs layers are denoted in bold. The waveguide from the bottom side was formed by a low doped InP substrate and from the top by 2.5 μm AlInAs layer covered by heavily doped InGaAs layer. The details of the layer structure of grown devices are listed in Table 4.

In order to evaluate the effect of optimization of growth procedure of InAlAs waveguide on laser performance, we have grown two QCL structures. The first QCL structure was grown employing the optimized growth conditions of the InAlAs waveguide, defined as T_g_ = 480 °C and V/III = 26, while the active region of the structure was grown at T_g_ = 520 °C and V/III = 12. The second QCL structure was grown according to previously developed technology, i.e., at constant temperature T_g_ = 520 °C and V/III = 12 [6].

**Figure 10:**
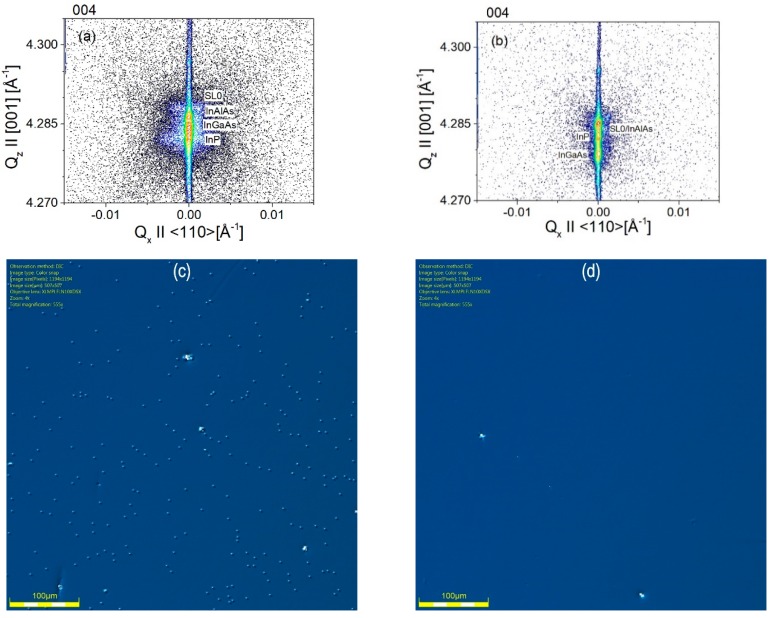
Reciprocal space map of X-ray intensity around 004 Bragg reflection for QCL sample grown at constant temperature T_g_ = 520 °C and V/III = 12 (**a**) and at the optimized growth technology (**b**). Surface morphology for sample grown at constant temperature T_g_ = 520 °C and V/III = 12 (**c**) and the one grown by optimized growth technology (**d**).

Figure 10 shows reciprocal space maps of X-ray intensity around 004 Bragg reflection for QCL samples grown in the above conditions. It is clearly seen that samples grown at optimized conditions show much less diffuse scattering and mirror like surfaces.

**Figure 11:**
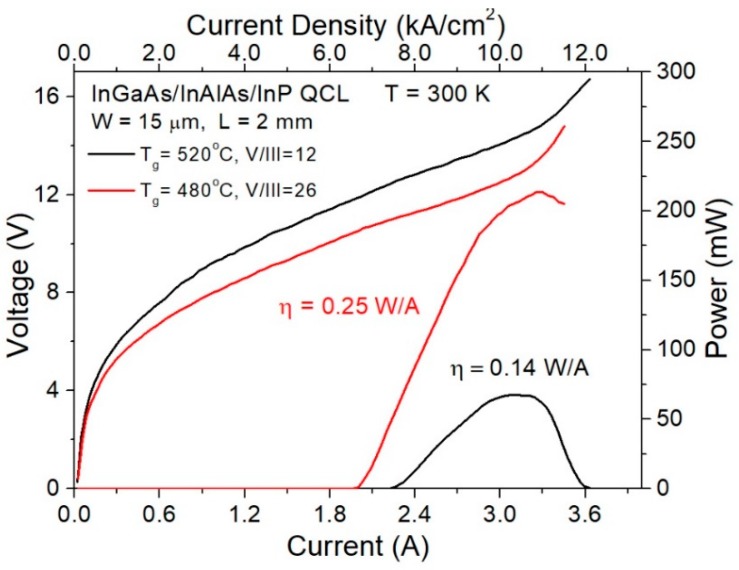
The room temperature light-current and voltage-current characteristics of two lattice-matched In_0.53_Ga_0.47_As/In _0.52_Al_0.48_As/InP lasers; the one grown according to optimized growth technology and another grown at constant temperature T_g_ = 520 °C and BEP ratio V/III = 12.

The structures grown were processed into double trench Fabry–Perot lasers using standard processing technology [31,32]. For the isolation layer, Si_3_N_4_ was used. Low resistivity electrical contacts were alloyed at 370 °C for 60 s. The Ti/Pt/Au alloy was used to the epi-side, and AuGe/Ni/Au for the low doped substrate side. The lasers were cleaved into bars with a length of 2 mm and mounted epi-side up on an Au-plated AlN submount. Figure 11 shows comparison of the room temperature electro-optical characteristics of the laser grown according to optimized growth technology and the one grown at constant temperature T_g_ = 520 °C and V/III = 12. QCL grown at optimized conditions exhibits a threshold current density of 6.67 kA/cm^2^, compared to 7.53 kA/cm^2^ for a laser grown at a constant temperature and almost twice improved differential quantum efficiency; 0.25 W/A versus 0.14 W/A. Threshold voltage for optimized QCL equals 10.2 V. Improved quantum efficiency is yet more proof of better optical quality of the InAlAs waveguide (lower scattering losses). The lasers emitted at 9.2 µm.

## 4. Conclusions

We have investigated molecular beam epitaxy growth conditions of micrometers-thick In_0.52_Al_0.48_As designed for a waveguide of InGaAs/InAlAs/InP quantum cascade lasers. The effects of growth temperature and V/III ratio on the surface morphology and defect structure were studied. The presented optimization of growth conditions of In_0.52_Al_0.48_As waveguide layer, based on the extensive X-ray diffraction studies, optical microscopy, AFM and photoluminescence, led to the growth of defect free material, with good optical quality. This has been achieved by decreasing the growth temperature of the InAlAs waveguide to T_g_ = 480 °C and increasing of the V/III ratio to 26. At the same time, the growth conditions of the cascade active region of the laser were left unchanged compared to our previous work; i.e., they stayed at T_g_ = 520 °C and V/III ratio equal to 12. The lasers grown using the new recipe have shown lower threshold currents and substantially improved slope efficiency. We relate this performance improvement to reduction of the electron scattering on the interface roughness and decreased waveguide absorption losses. The optimized epitaxy technology can be applied in many other optoelectronic devices, e.g., recently developed flexible microscale LEDs fabricated by a simple monolithic process [33].

## Figures and Tables

**Table 1:** Schematic of test samples structure used to study In_0.52_Al_0.48_As growth optimization versus the parameters of MBE: substrate temperature and V/III BEP ratio.

Thickness	Material	Doping
20 nm	In_0.53_Ga_0.47_As	undoped
2.5 μm	In_0.52_Al_0.48_As	undoped
450 μm—Substrate	InP:S	n = 3 × 10^18^ cm^−3^

**Table 2:** The relative decrease of reflectance at probe wavelength of 633.1 nm at the end of the growth process of 2.5-μm In_0.52_Al_0.48_As layer for all investigated substrate temperatures and V/III ratios.

Substrate Temperature	BEP Ratio			
	V/III = 31	V/III = 26	V/III = 17	V/III = 12
520 °C	−8.90%	−5.45%	−6.04%	−8.11%
500 °C	−1.94%	−3.59%	−2.84%	−2.43%
480 °C	−0.51%	−0.15%	−0.12%	−0.03%
460 °C	−11.45%	−3.25%	−6.03%	−5.80%

**Table 3:** The FWHM of the near band edge PL signal for 2.5-μm In_0.52_Al_0.48_As layer for all investigated substrate temperatures and V/III ratios.

Substrate Temperature	BEP Ratio			
	V/III = 31	V/III = 26	V/III = 17	V/III = 12
520 °C	19.0 nm	12.3 nm	10.9 nm	12.6 nm
500 °C	9.5 nm	9.8 nm	9.8 nm	9.9 nm
480 °C	9.3 nm	9.8 nm	9.8 nm	9.9 nm
460 °C	19.3 nm	42.7 nm	23.6 nm	52.0 nm

**Table 4:** Layer structure of lattice matched In_0.53_Ga_0.47_As/In_0.52_Al_0.48_As/InP laser.

Thickness	Material	Doping	Specification
500 nm	InGaAs	n = 8 × 10^18^ cm^−3^	Cap
2.5 µm	AlInAs	n = 1 × 10^17^ cm^−3^	Upper Waveguide
500 nm	InGaAs	n = 4 × 10^16^ cm^−3^	
**~1.8 μm**	**30 × InGaAs/InAlAs**		Active Region (AR)
500 nm	InGaAs	n = 4 × 10^16^ cm^−3^	Lower Waveguide
500 µm—Substrate	InP	n = 2 × 10^17^ cm^−3^	
